# Bacteraemia in patients admitted to an urban hospital in West Africa

**DOI:** 10.1186/1471-2334-7-2

**Published:** 2007-01-26

**Authors:** Philip C Hill, Charles O Onyeama, Usman NA Ikumapayi, Ousman Secka, Samuel Ameyaw, Naomi Simmonds, Simon A Donkor, Stephen R Howie, Mary Tapgun, Tumani Corrah, Richard A Adegbola

**Affiliations:** 1 Bacterial Diseases Programme, Medical Research Council Laboratories, Banjul, The Gambia; 2 The Royal Free and University College Medical School, London, UK

## Abstract

**Background:**

Few studies on bacteraemia in Africa have been published. We aimed to prospectively identify the causative organisms of bacteraemia in The Gambia and their relation to clinical diagnoses, outcome and antimicrobial susceptibility.

**Methods:**

Between November 2003 and February 2005 we studied those admitted to the Medical Research Council hospital who were suspected of having bacteraemia. We documented clinical features, outcome, pathogens identified and their susceptibility patterns, and searched for factors associated with bacteraemia.

**Results:**

871 patients were admitted and had a blood culture taken. The median age was 2 years (range 2 months to 80 years) and 36 of 119 tested were HIV positive; 54.5% were male. 297 (34%) had a positive result and 93 (10.7% overall) were considered a genuine pathogen. Those with bacteraemia were more likely to die in hospital (OR 2.79; 1.17–6.65, p = 0.017) and to have a high white cell count (WCC; OR 1.81;95% CI 1.09–3.02; p = 0.022). Three organisms accounted for 73% of bacteraemias: *Streptococcus pneumoniae *(45.2%), *Staphylococcus aureus *(18.3%) and *Escherichia coli *(9.7%) while non-typhoidal salmonellae (NTS) accounted for 8.6%. Antimicrobial susceptibility of *S. pneumoniae *was very high to penicillin (97.5%); high resistance was found to co-trimoxazole. *S. aureus *was generally highly susceptible to cloxacillin, gentamicin and chloramphenicol. *E. coli *and NTS were all susceptible to ciprofloxacin and mostly susceptible to gentamicin. Thirteen (33%) *S. pneumoniae *isolates were of serotypes contained in a 7-valent pneumococcal conjugate vaccine and 20 (51.3%) were of the same serogroup.

**Conclusion:**

In The Gambia, those with bacteraemia are more likely than those without to die in hospital and to have a raised peripheral blood WCC. *S. pneumoniae* is the most common organism isolated. Introduction of a pneumococcal conjugate vaccine can be expected to lead to a reduction in disease incidence.

## Background

There is a large excess of mortality in sub-Saharan Africa, particularly in children: under 5 year old mortality rates of 100–250 per 1,000 compare with 10–30 per 1,000 in developed countries [1]. It is increasingly clear that invasive bacterial infections are a major contributor to this excess [2], with incidence rates confirmed to be much higher than those reported in developed countries [3]. However there is a paucity of information as to the relative contribution of different organisms to bacterial infections in sub-Saharan Africa and how this varies across a full range of age groups.

The virtual elimination of *Haemophilus influenzae *type b (Hib) disease in The Gambia [4] has changed the relative importance of other pathogens in the etiology of invasive bacterial infection. It is thought that *Streptococcus pneumoniae *is probably the predominant organism, as indicated by the success of a 9-valent pneumococcal conjugate vaccine in children, which included an overall mortality benefit [5]. We prospectively studied patients admitted to the Medical Research Council (MRC) hospital in Greater Banjul (the opposite end of the country to the location of the pneumococcal vaccine trial) who were suspected of having bacteraemia, to identify the causative organisms in relation to clinical diagnoses and their antimicrobial susceptibility patterns, and to identify differences between those with bacteraemia and those without.

## Methods

### Setting

The Gambia is situated 12° north of the equator and has a limited rainy season from June to October and most malaria transmission occurs from July to November. The MRC in The Gambia has a 42-bed ward which serves as a primary and tertiary health facility for a mainly urban population. It does not provide any surgical, obstetric or neonatal care. Such patients are referred to the Royal Victoria Teaching Hospital in Banjul, which has facilities to manage them.

### Participants

We conducted a study to assess clinical and bacteriological characteristics of bacteraemia episodes in patients admitted at the MRC ward from November 2003 to February 2005. The decision to take blood samples for culture rested solely on the attending physician's clinical impression and judgment. Verbal consent was obtained prior to blood sampling. The study was approved by the joint Gambia government-MRC ethics committee.

### Data gathering

Case notes of all patients admitted were reviewed daily to identify those who had blood cultures as part of their routine workup. We developed, piloted and finalised a standardized questionnaire to record socio-demographic characteristics, clinical symptoms, tobacco and alcohol use, prior antibiotics, steroid and immunosuppressive agents use, history of concurrent medical conditions (e.g. diabetes, cancer, HIV, sickle cell disease), anthropometric and physical signs and the results of investigations. When antibiotics were prescribed, the name, route and time of administration were noted. The clinical diagnosis and in-hospital outcome were recorded. An infectious diseases physician confirmed the final diagnosis for each case after review of the respective form. The decision to request any investigation was based solely on the attending physician's clinical judgment and each patient received routine medical care.

### Blood cultures

Bacterial isolates were obtained from blood using an automated blood-culture system BACTEC 9050, (Becton Dickinson, Temse, Belgium). Commercially produced BD BACTEC™ PEDS PLUS™/F culture vials were used for specimens obtained from children aged one month to 15 years and BD BACTEC™ Plus Aerobic/F* and Plus Anaerobic/F* culture vials for specimens from adults aged >15 years by direct inoculation of culture media at the patient's bedside following manufacturer's instructions for quality control and blood volume requirements. Whenever in sufficient blood volume was obtained only the Plus Aerobic/F* vial was inoculated. Whenever there was shortage of commercial vials each of Tryptone Soya Broth (TSB) and Brain Heart Infusion (BHI) culture media were used for specimens from adults and TSB only was used for specimens from children. We used standard microbiological procedures as described previously [6]. Further identification was by cultural morphology and biochemical methods. In addition, serotyping of the pneumococcal isolates was performed with capsular and factor-typing sera (Statens Serum Institut, Copenhagen, Denmark) using an antibody coated latex agglutination assay. Antimicrobial sensitivity patterns were determined by Kirby-Bauer disk diffusion test using interpretative criteria described previously [6]. The microbiology laboratory submits to the external quality assurance programme of the United Kingdom National External Quality Assessment Service.

Isolates were classified as contaminants if they were known to be a common skin contaminant, if there was scanty growth not on the line of inoculum that failed to grow on subculture, or if the isolate was obtained from the TSB aerobic bottle only after both bottles (TSB and BHI) had been inoculated with blood.

Malaria slides were stained by Field's stain and examined using a ×100 oil immersion lens. Testing for HIV-1 or HIV-2 infection was by competitive enzyme linked immunosorbent assays (Wellcome Laboratories, Kent, UK) and Western blot (Diagnostics Pasteur, Marnes-la-Coquette, France).

### Data analysis

All data were double-entered into an ACCESS database and checked for errors. We compared those with bacteraemia with those without. Weight for age was standardized using the CDC 2000 growth reference [7]. We defined the wet season as from June to November and the dry season from December to May. We defined tachycardia as >= 160 Beats per minute in infants under 12 months of age, 140 in 1 to 5 year old children and 120 in those 6 years old and over. A raised respiratory rate was >50 breaths per minute in infants and >40 in other age groups. A clinical diagnosis of severe malnutrition was defined as either visible severe wasting or Kwashiokor. Septicaemia was defined as the presence of bacteraemia in the absence of a focus of infection. We assessed possible risk factors for bacteraemia by Chi-Square test and, in addition to age and sex that were included a priori, considered those with a p value of <= 0.15 for a multi-variable logistic regression model. The explanatory variables in the final adjusted model were age (continuous variable), sex, temperature (categorical variable) and white cell count (categorical variable). A separate model compared the outcome of those with bacteraemia to those without, adjusting for the above variables. Analyses were conducted using Stata software (version 8; Stata Corp, College Station, TX).

## Results

During the study period, 2712 patients were admitted to the MRC ward, the median age was 5 years (range 2 months to 80 years) and 871 underwent phlebotomy for blood culture. The median age of these patients was 2 years (range 2 months to 80 years) and 54.5% were male (table 1). Nine patients had taken corticosteroids in the last month, one had a history of malignancy, 6 were known to be diabetic. Two hundred and forty nine patients had a screening test for sickle cell disease, 58 (23.3%) were positive. The median duration of stay was 4 days (range: 1 to 45 days). Forty nine (5.5%) patients died in hospital. The overall mortality in the hospital was 6% during the period. One hundred and nineteen patients had known HIV status or were tested during the admission because of one or more clinical indication; 36 (30.2%) were HIV positive.

**Table 1:** Characteristics of the patients admitted with suspected bacteraemia.

**Characteristics**	**All (n = 871)**	**Bacteraemia (n = 93)**	**non-Bacteraemia (n = 574)**	**p value**
**Age**				
2 mo–11 mo	200 (23.0)	24 (25.8)	110 (19.2)	
11 mo–23 mo	207 (23.8)	18 (19.4)	127 (22.1)	
2–5 years	166 (19.1)	23 (24.7)	112 (19.5)	
6–15 years	113 (13.0)	13 (14.0)	77 (13.4)	
>15 years	185 (21.2)	15 (16.1)	148 (25.8)	0.185
**Male**	475 (54.5)	59 (63.4)	312 (54.4)	0.102
**Wet season admission**	533 (61.2)	60 (64.5)	405 (70.6)	0.295
**Duration of symptoms < 7 days (n = 849)**	459 (54.1)	43 (47.8)	307 (55.1)	0.252
**Weight for age z score <-2 (n = 693)**	373 (53.8)	39 (51.3)	221 (51.0)	0.87
**Temperature >= 38°C (n = 863)**	364 (42.2)	48 (51.6)	229 (40.3)	0.041
**Tachycardia (n = 738)**	253 (34.2)	38 (46.9)	182 (37.3)	0.141
**Raised respiratory rate (n = 677)**	347 (51.3)	51 (62.2)	247 (55.2)	0.297
**White Blood Cell Count (n = 852)**				
>= 10 × 10^9^/L	484 (56.8)	64 (70.3)	306 (54.6)	0.005
>= 15 × 10^9^/L	290 (34.0)	45 (49.5)	173 (30.8)	<0.0001
**Haemoglobin < 8 g/L (n = 602)**	192 (32.0)	30 (41.1)	121 (31.8)	0.121
**Sickle cell test positive (n = 243)^a^**	57 (6.5)	8 (8.6)	13 (2.2)	0.144
**Died in hospital**	49 (5.5)	8 (8.3)	25 (4.3)	0.081
**Specific Clinical Diagnoses**				
Pneumonia	332 (38.1)	40 (43.0)	207 (36.1)	0.198
Malaria (n = 676 tested)^a^	101 (11.6)	10 (10.8)	65 (11.3)	0.82
Severe Malnutrition	68 (7.8)	8 (8.6)	50 (8.7)	0.975
Tuberculosis	34 (3.9)	1 (1.1)	29 (5.1)	0.086
HIV/AIDS (n = 119 tested)^a^	36(4.1)	6 (6.4)	25 (4.4)	0.397
Gastroenteritis	36 (4.1)	1 (1.1)	24 (4.2)	0.145
Urinary tract infection	25 (2.9)	2 (2.2)	19 (3.3)	0.573
Nephrotic/Nephritic syndrome	23 (2.6)	1 (1.1)	17 (3.0)	0.297
Skin/soft tissue infection	20 (2.3)	3 (3.2)	15 (2.6)	0.74
Meningitis	13 (1.5)	1 (1.1)	9 (1.6)	0.716
Upper respiratory tract infection	12 (1.4)	0	9 (1.6)	0.252
Hepatititis	12 (1.4)	0	10 (1.7)	0.235
Bone/Joint infection	9 (1.0)	0	6 (1.0)	0.387

^a ^For sickle cell, malaria and HIV tests, because the investigations were driven by clinical suspicion, the denominator for the % calculations remained the total number in each category.

**Figure 1:**
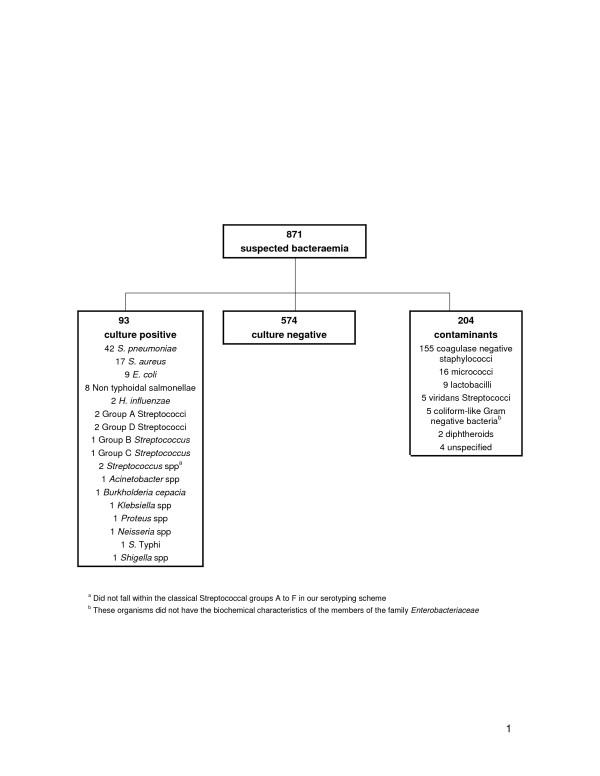
Flow diagram of suspected bacteraemia patients according to blood culture results.

Of those who had a blood culture taken, 297 (34%) had a positive result and 93 (10.7% overall) were considered to be a genuine pathogen (Figure 1). Those whose blood culture grew a contaminant were excluded from further analyses because of the possibility of masking of a true bacteraemia. Of those with bacteraemia, the same organism was obtained from another normally sterile site in 12 (12.9%) cases.

Those with bacteraemia were more likely to die in hospital and, while this was not significant by Chi-Square, in an adjusted model it was significant (OR 2.74; 95% CI 1.15–6.53; p = 0.022). Those with bacteraemia were more likely to have a temperature >= 38°C, although in the adjusted model this lost significance (OR 1.42; 95% CI 0.9–2.25; p = 0.14), having been subject to confounding by WCC. Those with a WCC of at least 10 × 10^9^/L at presentation were also more likely to be bacteraemic, remaining significant in the adjusted model (OR 1.81;95% CI 1.09–3.02; p = 0.022). For those with a WCC of at least 15 × 10^9^/L, the adjusted OR was 1.97 (95% CI 1.23–3.15; p = 0.005). Almost 40% of those in the study had a clinical diagnosis of pneumonia and the distribution of clinical diagnoses were similar between the two groups. Of those with pneumonia 276 (83%) had an xray, and 263 (95.3%) were assessed as being abnormal.

Three organisms combined to account for 73% of all bacteraemias (table 2); *S. pneumoniae *(45.2%), *Staphylococcus aureus *(18.3%) and *Escherichia coli *(9.7%) while non-typhoidal salmonellae(NTS) accounted for 8.6% of isolates. Of note, *H. influenzae *accounted for 2 isolates (1 Hib and 1 Hi non-serotypable) and *Neisseria *spp. accounted for 1 isolate (Figure 1). None of the organisms detected were more likely to be identified in the wet season. In those who were bacteraemic, 2 of 10 with malaria on blood film had NTS as opposed to 4 of 70 who had a negative blood film, although this difference was not significant (p = 0.12). *S. pneumoniae *and *S. aureus *were relatively evenly distributed across the age groups. In contrast, 5 of the 9 *E. coli *isolates were found in infants, although only one was under 6 months of age. Of the 8 (8.6%) bacteraemic patients who died, 3 had *S. pneumoniae*, 2 had *S. aureus*, 2 had NTS and 1 had *Proteus *spp.

**Table 2:** Number of isolates/groups of isolates and their relative proportion (%) in each of 5 age bands and in each of 6 clinical diagnostic categories for those with Bacteraemia (n = 93).

	**Organism/group, n (% of row)**					
	**S. pneumoniae**	**S. aureus**	**Other Gram +ve**	**E. coli**	**NT^a ^salmonellae**	**Other Gram -ve**
**Age group**						
2 mo–11 mo	9 (37.5)	2 (8.3)	4 (16.7)	5 (20.8)	2 (8.3)	2 (8.3)
11 mo–23 mo	8 (44.4)	4 (22.2)	1 (5.6)	1 (5.6)	2 (11.1)	2 (11.1)
2–5 years	12 (52.2)	4 (17.4)	1 (4.4)	3 (13.0)	1 (4.4)	2 (8.7)
6–15 years	7 (53.4)	3 (23.1)	1 (7.7)	0	0	2 (15.4)
>15 years	6 (40.0)	4 (26.7)	1 (6.7)	0	3 (20.0)	1 (6.7)
All	42 (45.2)	17 (18.3)	8 (8.6)	9 (9.7)	8 (8.6)	9 (9.7)
**Diagnostic category**						
Pneumonia	30 (78.9)	4 (10.5)	2 (5.3)	0	0	2 (5.3)
Sepsis	7 (29.2)	6 (25.0)	3 (12.5)	2 (8.3)	3 (12.5)	3 (12.5)
Malaria	0	1 (14.3)	0	2 (28.6)	2 (28.6)	2 (28.6)
HIV/AIDS	1 (16.7)	2 (33.3)	0	1 (16.7)	2 (33.3)	0
Severe Malnutrition	2 (40.0)	1 (20.0)	2 (40.0)	0	0	0
Other	2 (15.4)	3 (23.1)	1 (7.7)	4 (30.8)	1 (7.7)	2 (15.4)

^a ^Non-typhoidal

Of 39 isolates of *S. pneumoniae *that were characterised further by serotyping, 14 serotypes/groups (1,5,14,23F,6A,19A,6B,,4,9V,9L,10,15,18C,22) were identified, two (1 and 5) accounted for 16 (41%) of the isolates. Thirteen (33%) were of serotypes contained in a 7-valent pneumococcal conjugate vaccine (4, 6B, 9V, 14, 18C, 19F, and 23F) and 20 (51.3%) were of a 7-valent vaccine serogroup.

Isolates of *S. pneumoniae *(39, 97.5%) were highly susceptible to penicillin (Table 3), ampicillin (100%) and chloramphenicol (97.6%). They were moderately susceptible to tetracycline (65.9%) and only poorly susceptible to co-trimoxazole (5%). *S. aureus *isolates were all susceptible to cloxacillin, gentamicin and chloramphenicol; moderately susceptible to co-trimoxazole (66.7%) and poorly susceptible to tetracycline (33%) and penicillin (8%). Among Gram-negative bacteria, all isolates of *E. coli *(9) and NTS (5–7%) were susceptible to ciprofloxacin. In addition, *E. coli *isolates were highly susceptible to gentamicin (88.9%) and moderately susceptible to chloramphenicol (66.7%), poorly susceptible to co-trimoxazole (22%) and completely non-susceptible to ampicillin. Non-typhoidal isolates were also susceptible to gentamicin (100%), tetracycline (80%), co-trimoxazole (71%), chloramphenicol (66.7%) and ampicillin (57%).

**Table 3:** Antibiotic susceptibility of the 3 major organisms isolated and non-typhoidal salmonellae in relation to the number tested.

**Organism**	**n susceptible/n tested**							
	Penicillin	Ampicillin	Cloxacillin	Cotrim	Gent	Chloram	Tetra	Cipro
*S. pneumoniae*	39/40	42/42	ND	2/39	ND	40/41	27/41	ND
*S. aureus*	4/14	ND	14/14	10/15	15/15	15/15	5/15	ND
*E. coli*	ND	0/9	ND	2/9	8/9	6/9	ND	9/9
NT^a^-salmonellae	ND	4/7	ND	5/7	7/7	4/6	4/5	7/7

^a ^Non-typhoidal

## Discussion

In this study we have shown that Gambian patients with bacteraemia are more likely than those without to die in hospital and to have a raised peripheral blood WCC. Three organisms accounted for 73% of all bacteraemias; *S. pneumoniae *accounted for 45%. This hospital based study emphasizes the dominance of *S. pneumoniae *in the etiology of bacteraemia in The Gambia, consistent with previous etiology studies in this country [8-10].

*S. pneumoniae *has been identified as the dominant isolate in bacteraemia elsewhere in Africa. Berkowitz [11] identified bacteraemia in 315 (5.8%) of 5397 children admitted to a South African hospital; 23% died. *S. pneumoniae *accounted for 23% of isolates, *S. aureus *6%, *Salmonella *species 20%, *E. coli *13%, and *H. influenzae *14%. Cotton et al [12] documented 132 episodes of community-acquired bacteraemia in hospitalized South African children; 12% died. *S. pneumoniae *accounted for 33% of pathogens isolated, *S. aureus *14%, *N. meningitidis *11% and other Gram negative organisms 18%. Berkley et al [3] found that 866 (5.9%) of 14,787 children over 60 days of age admitted to a rural hospital in Kenya had bacteraemia. *S. pneumoniae *accounted for 30% of isolates, *S. aureus *7%, other Gram positive cocci 6%, NTS 19%, *H. influenzae *15%, and *E. coli *10%. In Malawi, Archibald et al [13] found blood stream infection in 70 (30%) of 233 febrile adults admitted to hospital, 70% of whom were HIV positive. *S. pneumoniae *accounted for 33% of isolates, *Salmonella *species 19% and Gram positive cocci only 4%. *M. tuberculosis *accounted for 29% of isolates, found exclusively in HIV positive patients.

*Salmonella *species have predominated in several other African studies, particularly in high HIV prevalence settings. Bahwere et al [14] identified 126 (15.9%) bacteraemias in 779 children admitted to a hospital in Congo;19.4% died. *Salmonella *species accounted for 44% of isolates. Gram positive bacteria accounted for only 10% of isolates. Ghiorghis et al [15] identified bacteraemia in 49 (7.7%) of 634 febrile children at an Ethiopian hospital. Of the organisms isolated, *Salmonella *species accounted for 57%, streptococci 16%, staphylococci 14%, and *E. coli *6% and other Gram negative species 6%. Walsh et al [16] reported that of 365 positive isolated from children in Malawi, NTS accounted for 38% of isolates, *H*. influenzae 6%, other Gram negative species 29%, *S. pneumoniae *16%, other streptococci 8% and *S. aureus *2%. Gordon et al [17] identified 449 pathogens from the blood of adult patients in Blantyre: NTS accounted for 37% of isolates and 30% were *S. pneumoniae*. NTS accounted for 70–80% of all paediatric blood culture isolates in a hospital in Western Zaire [18]. *S. aureus *was identified by Meremikwu et al [19] as the cause of bacteraemia in 48.7% of bacteraemias in children admitted to a Nigerian hospital, 44% were neonates. Differing criteria for patient selection, variation in the standardisation of methods and bacteriological techniques used may explain some of the differences seen between studies.

Others have identified risk factors for bacteraemia in African settings. Ghiorghis et al [15] found no difference in nutritional status, age or temperature between febrile bacteraemic cases and controls, but did find increased WCC in those with salmonellae and in those with *E. coli*. Bahwere et al [14], in their study dominated by *Enterobacteraciae*, also found no association with severe malnutrition. However, Cotton et al [12], Berkowitz et al [11] and Friedland [20] identified severe malnutrition as a risk factor for bacteraemia and death in South Africa. Archibald et al [13] identified HIV positivity as a risk factor for bacteraemia in Malawi. HIV and malnutrition have been shown to be independent risk factors for bacteraemia by Berkley et al [3].

In contrast to our study, seasonal variation in the etiology of bacteraemia has been demonstrated in sub-Saharan Africa. Bell et al [21], in a Malawi population with high rates of HIV positivity, found that NTSpredominated in the wet season while *S. pneumoniae *predominated in the dry season. High prevalence of NTS in the wet season in sub-Saharan Africa is thought to be associated with malaria [22-24], although other, often site-specific, factors should be considered as well. We had too few cases of NTS to assess seasonal variation properly in this study.

Other studies from The Gambia confirm that high resistance to penicillin is not yet prevalent here [10,6]. In contrast, high resistance was found to co-trimoxazole, which is widely used at primary health care level. Evaluation of an alternative antimicrobial for community management of non-severe bacterial infections is now underway. Although numbers of isolates were small, *S aureus *was in general highly susceptible to cloxacillin, gentamicin and chloramphenicol. Penicillin was the least effective antibiotic. Unlike several other developing countries where resistance has been reported to these antimicrobials, cheap and widely available antibiotics remain effective in The Gambia. A larger study to give more precise estimates of antimicrobial susceptibility of isolates of *E. coli *and NTS is required.

While this study represents 'real life' clinical practice in this hospital and our data were prospectively gathered, our approach has several limitations. A more formal study would set strict criteria for taking blood for culture and standardize all clinical and laboratory procedures. Such an approach would enable certain tests to be routine, such as HIV testing, and may involve sending certain isolates to a reference laboratory for further identification. It is estimated that 5–10% of inpatients in our hospital are HIV positive (unpublished data). Despite having a standardized form, we did not ask the clinician to record his/her primary reason for taking a blood culture (we felt this would be difficult to categorize), although 47% of patients had a temperature of 37.5°C or more and 13% of these had a pathogen isolated. As 80% of our study subjects were under 5 years of age, interpretation of our findings in the older age groups should be done cautiously. We also cannot calculate an incidence rate of community-acquired bacteraemia as the source population of this hospital cannot be clearly defined and we did not obtain blood cultures from a representative sample of outpatient attendees [25].

## Conclusion

This study confirms that bacteraemia is an important illness in hospital patients in The Gambia. It appears that the most effective way to combat common severe bacterial infections in Africa is through vaccination [26], a premise supported by the virtual elimination of Hib disease from The Gambia [4]. The success of the 9-valent pneumococcal conjugate vaccine against pneumococcal disease in The Gambia, [5] together with the results of the present study, dictate that the introduction of a pneumococcal vaccine into the routine vaccination schedule is a high priority. It is intended that the currently available 7-valent pneumococcal conjugate vaccine will be introduced into The Gambia. This vaccine could be expected to protect against a substantial proportion of invasive pneumococcal disease and may lead to improvements in resistance to some antibiotics. However, it will not prevent disease by pneumococci of serotypes 1 and 5, the most prevalent serotypes in invasive pneumococcal disease in The Gambia [6]. Therefore we will monitor the introduction of this vaccine with a surveillance system to identify trends in the incidence of invasive pneumococcal disease and changes in serotype prevalence [27].

## Competing interests

The author(s) declare that they have no competing interests.

## Authors' contributions

PH conceived the study and was involved in the design, clinical aspects, data analysis and wrote the paper with CO and RA, who were responsible for the clinical aspects and laboratory aspects of the study respectfully. SA participated in the clinical aspects of the study. OS coordinated the lab work with UI and RA. NS developed the form for the study and piloted and finalized it. SD managed the data entry and verification and quality. SH was involved in the design of the study, in particular the study form, and was involved in the clinical care of the children. MT supervised the clinical aspects of the study with TC. All authors read and approved the final manuscript.

## Pre-publication history

The pre-publication history for this paper can be accessed here:


